# Role of invasive and non-invasive additional imaging methods in pediatric rheumatology

**DOI:** 10.1186/1546-0096-12-S1-P201

**Published:** 2014-09-17

**Authors:** Aleksey Kozhevnikov, Nina Pozdeeva, Michael Konev, Aleksey Moskalenko, Arthur Bergaliev, Konsantin Afonichev

**Affiliations:** 1 Hospital Rheumatology and Orthopedic Department, The Turner Scientific and Research Institute for Children's Orthopedics, Saint-Petersburg, Russian Federation; 2 Department of X-ray Diagnostic, The Turner Scientific and Research Institute for Children's Orthopedics, Saint-Petersburg, Russian Federation

## Introduction

Juvenile arthropathy it’s a term combining heterogeneous group of joints diseases in children, which are characterized by structural, morphological and functional changes of inflammatory nature and non inflammatory disorders. Chronic inflammatory process may be "driving" mechanism that is determine the nature of the articular lesions with specific clinical, instrumental and laboratory picture or maybe just one of the features of another pathology. Similarity of clinical and radiological picture, chronic nature of the flow, various of pathogenetic therapy and outcomes of the disease do this group one of the difficult to diagnose.

## Objectives

To study the masks of JIA and no rheumatology pathology joint in children of Russian Federation.

## Methods

Clinical, serological, x-ray manifestations, ultrasound, MRI, arthroscopy data were analyzed in more then 300 children which examined in hospital department with presumptive diagnosis JIA.

## Results

All children were subjected to complete rheumatology examination, and remained under our observation for a long time in order to assess the dynamic of the articular lesion. Children with classic course of JIA were appointed basic anti-inflammatory drugs (methotrexat, anti-TNF, IL6 block). In cases when clinically atypical duration or laboratory "mute" of arthritis, long persistence monoarthritis and also variants with fever or destructive bones change we were applied deeper imaging methods such as MRI, arthroscopy, 3-phase osteo scintigraphy and other. Over five years were diagnosed six cases of tuberculous lesion of the joints (specific destruction of bone + biopsy), two cases of tuberculous periarthicular soft tissue (cyst formation with tyromatosis), fourteen cases of vascular anomaly (cavernous hemangioma, dysplasia of the superficial veins), five cases of atypical localization focus of osteoid-osteoma (the bottom of the acetabulum, the bottom of the fossa coronoidea humerous, processus posterior of the talus), six cases of pigmental villous and chronic hemorrhagic synovitis of the knee and ankle joint, several cases of the skeletal dysplasia, and in rare cases of histiocytosis, malignant B-cell lymphoma, osteomyelitis and other rare bones disorders. Not one cases of the CRMO. In many cases due to this methods, we were excluded such diagnosis like tumors of the joints, different forms osteochondropathy. Such typical changing of the bones in JIA are usually degenerative nature in chronic uncontrolled inflammation.

## Conclusion

In some cases in undifferentiated arthritis, time of the diagnosis may determine the success of treatment. Any arthritis after failure of anti-inflammatory and antibacterial therapy should be questioned of rheumatic nature. Application invasive and non-invasive additional imaging methods can reduce the risk of incorrect diagnosis.

## Disclosure of interest

None declared.

